# Supplementary material for: Conductive 2D metal-organic framework for high-performance cathodes in aqueous rechargeable zinc batteries
Source: Nat Commun. 2019 Oct 30;10:4948. doi: 10.1038/s41467-019-12857-4 (PMC6821766; doi:10.1038/s41467-019-12857-4)
Supplement: Supplementary file 1 — Supplementary Information [file 41467_2019_12857_MOESM1_ESM.pdf]

Supplementary Information

Conductive 2D metal-organic framework for high-performance cathodes in aqueous rechargeable zinc batteries

Nam, *et al*.

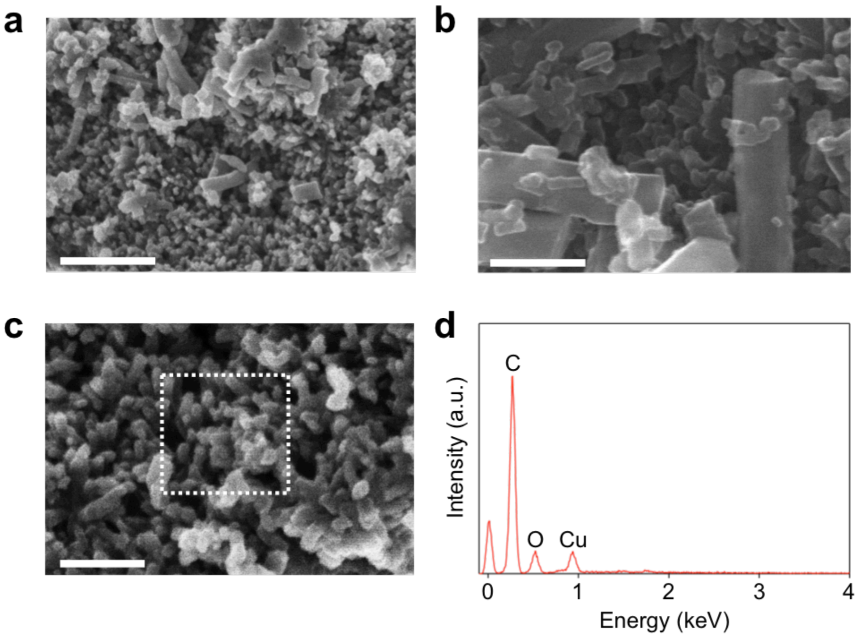

Supplementary Fig. 1 | SEM images of $Cu_3(HHTP)_2$ powder at **(a, b)** low and **(c)** high resolution. **(d)** EDX spectrum for the selected area in **c**. Scale bars in **a-c** are 500, 250, and 100 nm, respectively.

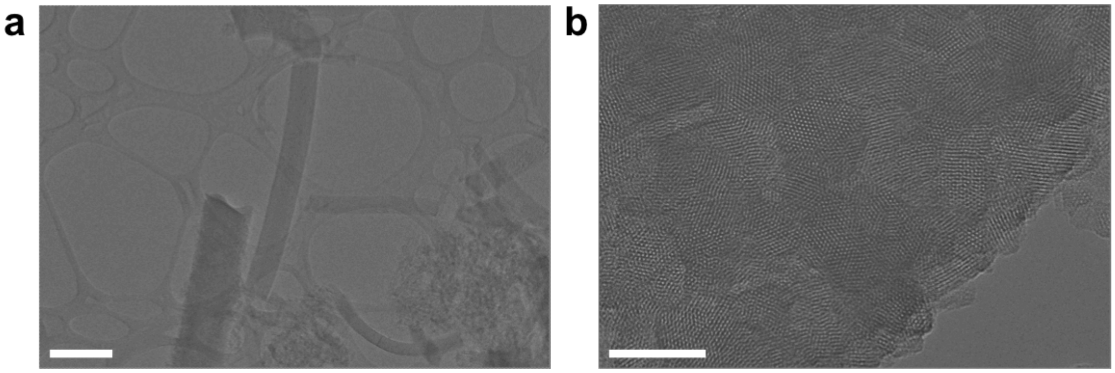

Supplementary Fig. 2 | TEM images of $Cu_3(HHTP)_2$ pristine at **(a)** low and **(b)** high magnification. Scale bars in **a, b** are 500 and 50 nm, respectively.

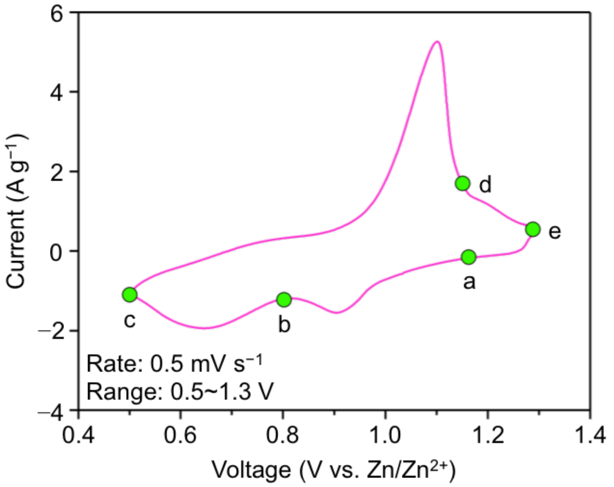

Supplementary Fig. 3 | Cyclic voltammogram of $Cu_3(HHTP)_2$. Cyclic voltammetry (CV) was performed using a coin-type cell, two-electrode configuration with active electrode composed of $Cu_3(HHTP)_2$: acetylene black : PVDF = 6 : 2 : 2. The green dots a−e mark the states where the XPS analysis was conducted.

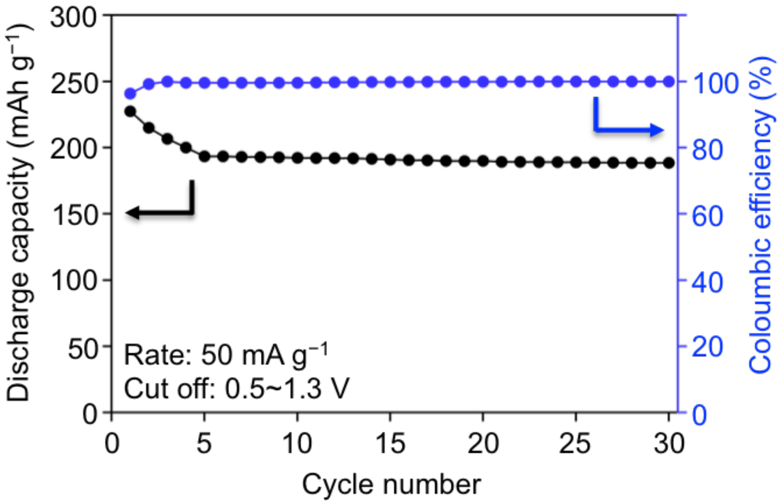

Supplementary Fig. 4 | Cycle performance of $Cu_3(HHTP)_2$ at a current density of 50 mA g^{-1}

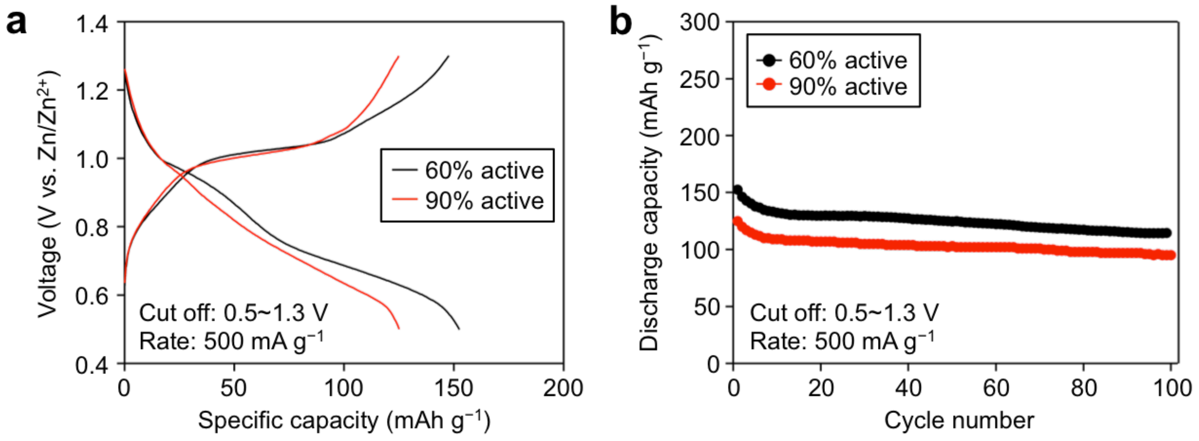

Supplementary Fig. 5 | (a) Discharge–charge voltage profiles and **(b)** cycling performance of $Cu_3(HHTP)_2$, depending on the mass loading of active materials at 500 mA g^{-1}. The electrode with 90% active materials was composed of $Cu_3(HHTP)_2$: acetylene black : PVDF = 90 : 5 : 5.

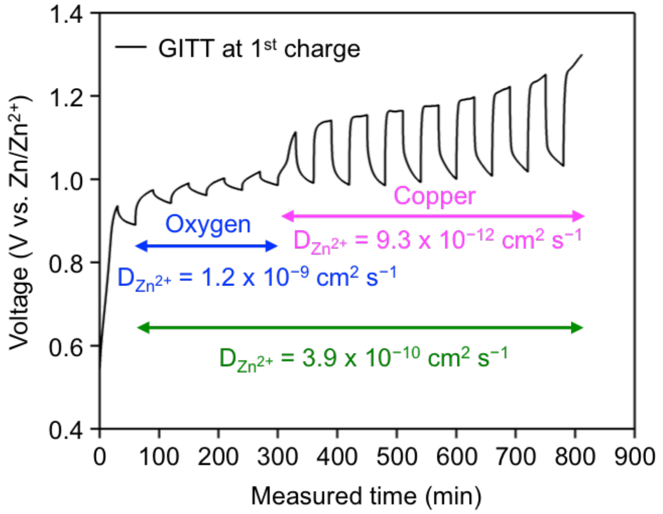

Supplementary Fig. 6 | A GITT curve of $Cu_3(HHTP)_2$ for the first charge process between 0.5 V and 1.3 V (current density: 50 mA g^{-1}, time interval τ: 30 min)

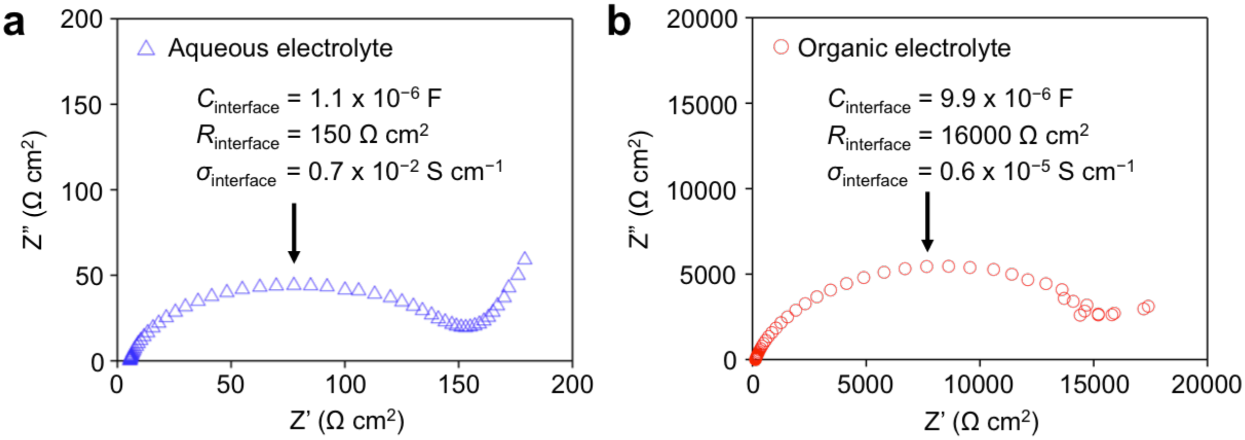

Supplementary Fig. 7 | EIS spectra for $Cu_3(HHTP)_2$ electrodes in **(a)** an aqueous and **(b)** an organic electrolyte. 3 M $Zn(CF_3SO_3)_2$ in DI H_2O and 0.25 M $Zn(CF_3SO_3)_2$ in MeCN were used as the electrolytes for aqueous and organic electrolytes, respectively.

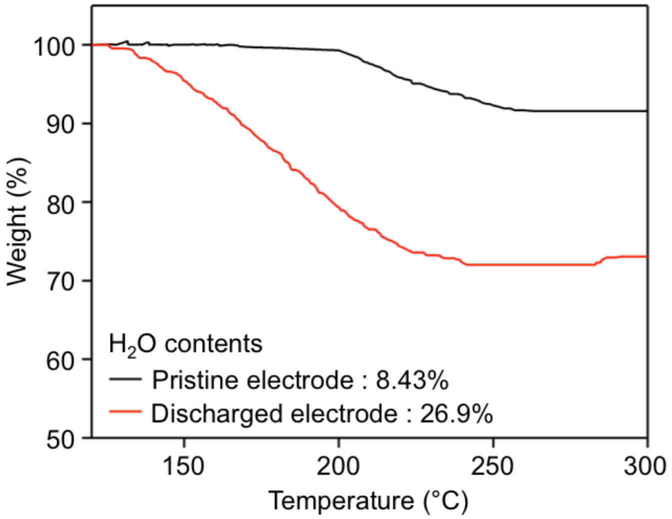

Supplementary Fig. 8 | TGA profiles of $Cu_3(HHTP)_2$ electrodes at the pristine and the discharged states

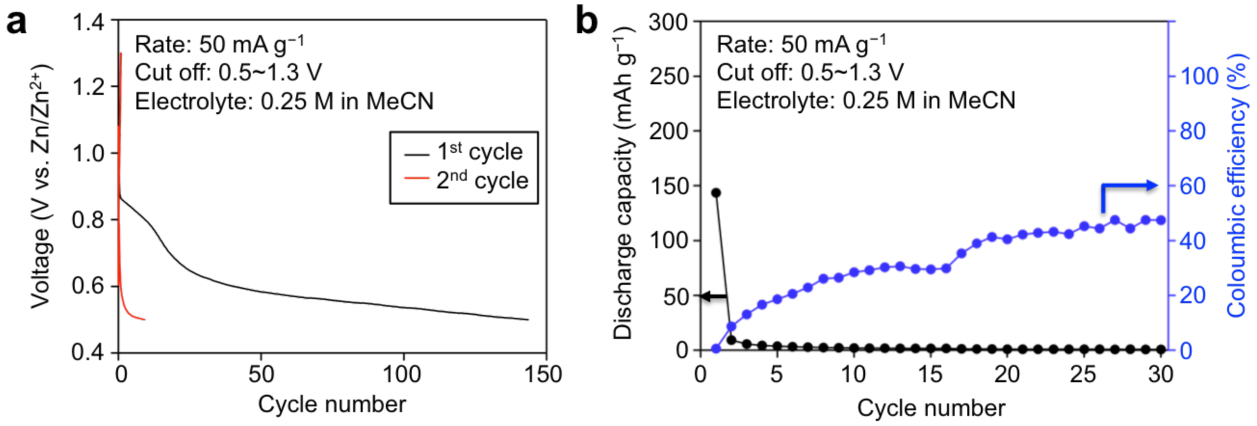

Supplementary Fig. 9 | (a) Discharge–charge voltage profiles and **(b)** cycling performance of $Cu_3(HHTP)_2$ with 0.25 M $Zn(CF_3SO_3)_2$ in MeCN at 50 mA g^{-1}

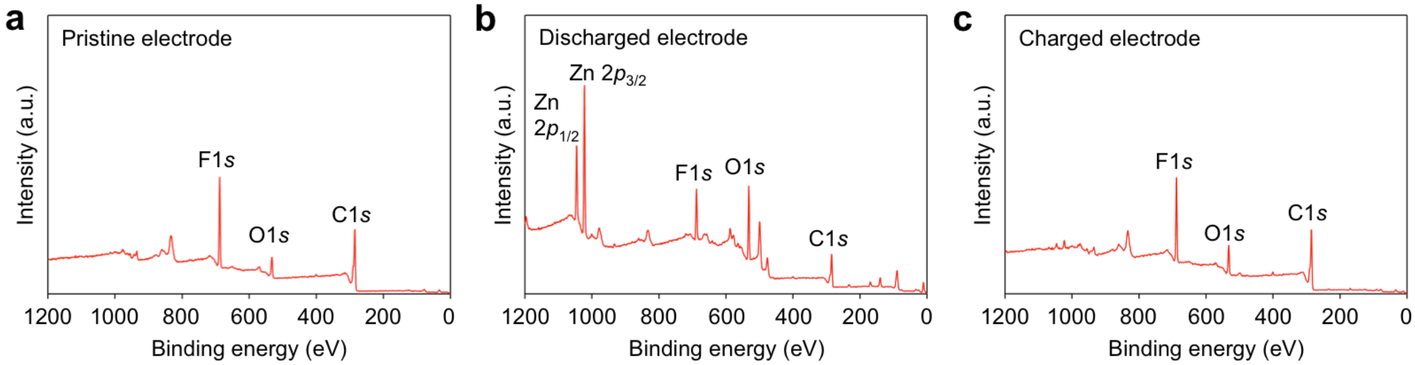

Supplementary Fig. 10 | Ex situ XPS survey spectra of $Cu_3(HHTP)_2$ at **(a)** pristine, **(b)** discharged, and **(c)** charged electrodes

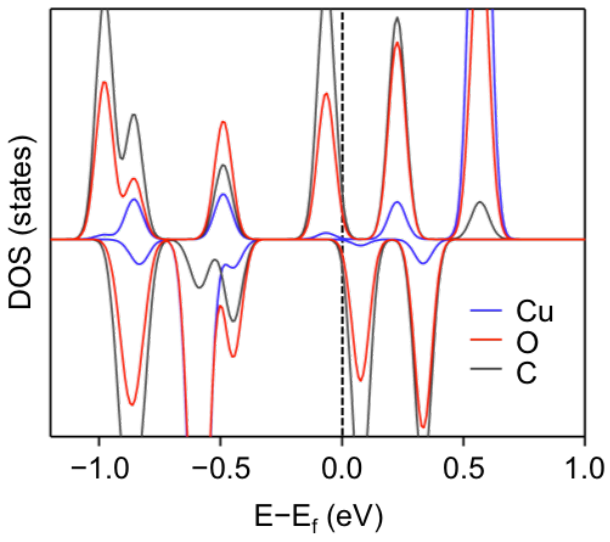

Supplementary Fig. 11 | Density of states near the Fermi level of the $Cu_3(HHTP)_2$ monolayer

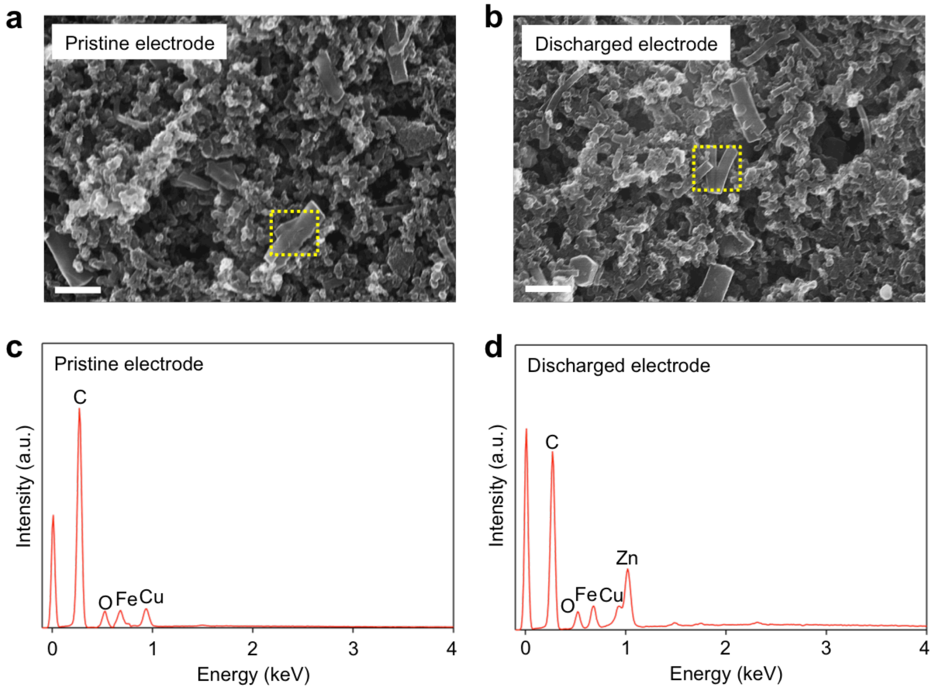

Supplementary Fig. 12 | SEM Images of **(a)** pristine and **(b)** discharged electrode composed of $Cu_3(HHTP)_2$: acetylene black : PVDF = 6 : 2 : 2. Scale bars in **a, b** are 500 nm. EDX spectra for **(c)** the pristine electrode in the selected area in **a** and **(d)** the discharged electrode in the selected area in **b**

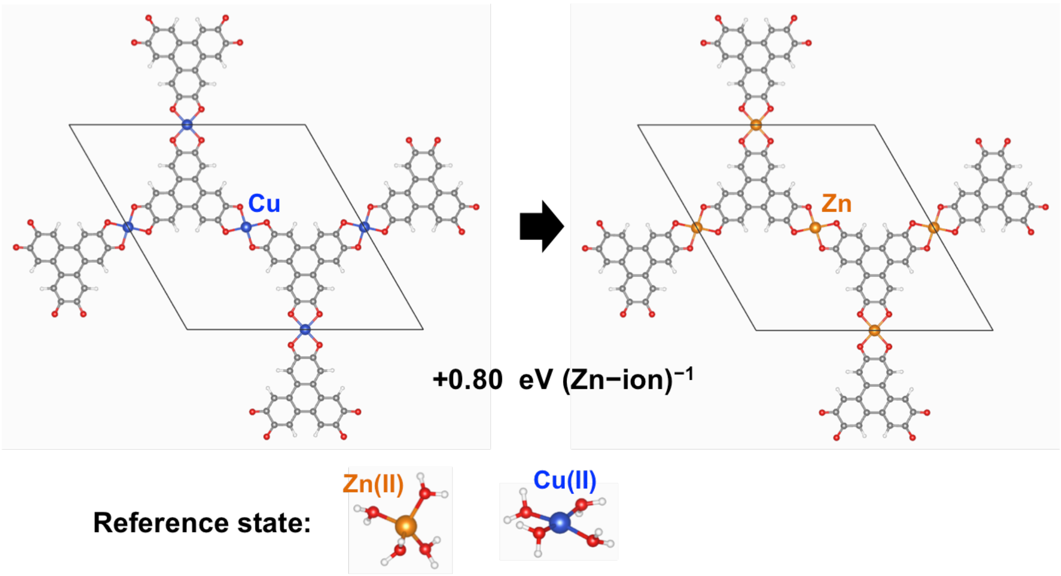

Supplementary Fig. 13 | Changes of relative energy after substituting Cu^{2+} to Zn^{2+} ions in $Cu_3(HHTP)_2$. As a reference state for energy comparison, respective hydrated divalent cations with an implicit solvent model were used.

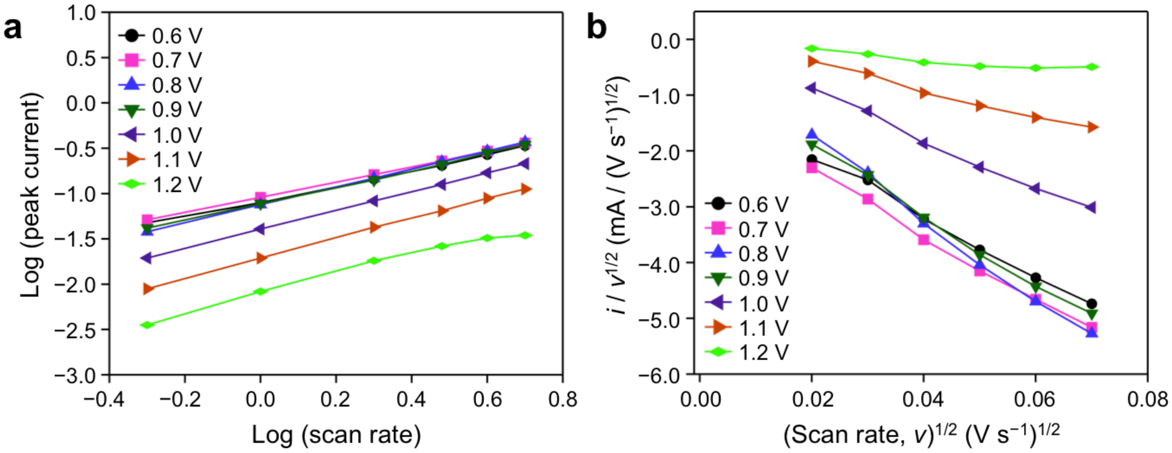

Supplementary Fig. 14 | (a) Peak currents as a function of scan rate in the range of 0.6–1.2 V during reduction process. **(b)** Use of Supplementary Equation 3 to analyze the capacitance currents of $Cu_3(HHTP)_2$ electrode. The solid lines are visual guides to the experimental data points.

Supplementary Table 1 | Comparison of rate performances of $Cu_3(HHTP)_2$ with reported Prussian Blue analogue cathodes with high rate capabilities.

**Cathodes**	**Operating Voltage (V vs. Zn/Zn^{2+})**	**Specific Capacity**	**Ref.**
$Cu_3(HHTP)_2$	0.97	228.0 mAh g^{-1} at 50 mA g^{-1}<br>124.0 mAh g^{-1} at 4000 mA g^{-1}	This work
ZnHCF	1.70	65.4 mAh g^{-1} at 60 mA g^{-1}<br>32.3 mAh g^{-1} at 1200 mA g^{-1}	1
C-RZnHCF	1.73	66.5 mAh g^{-1} at 60 mA g^{-1}<br>29.3 mAh g^{-1} at 1200 mA g^{-1}	2
CuHCF	1.73	55.0 mAh g^{-1} at 60 mA g^{-1}<br>42.5 mAh g^{-1} at 600 mA g^{-1}	3

Supplementary Note 1: The chemical diffusion coefficient

The chemical diffusion coefficient ($D_{Zn^{2+}}$) presented here is the average value of all D calculated in each interval according to Equation (Supplementary Equation 1, below), which was originally derived by Weppner and Huggins[4].

$$D = \frac{4}{\pi\tau}\left(\frac{m_B V_M}{M_B S}\right)^2 \left(\frac{\Delta E_s}{\Delta E_t}\right)^2 \quad (\tau \ll L^2/D) \qquad \text{(Supplementary Equation 1)}$$

τ : time for an applied galvanostatic current I_0

m_B: mass of active material

V_M: molar volume of active material

M_B : molecular weight of active material

S : interface area between the active material and electrolyte

ΔE_s : steady-state (equilibrium) voltage

ΔE_t : total change of the cell voltage E during the current pulse

L : radius of the active particle

Supplementary Note 2: Overall Reaction Formula of Zn-$Cu_3(HHTP)_2$ Battery

The initial reversible capacity of $Cu_3(HHTP)_2$ is 228 mAh g^{-1} at a rate of 50 mA g^{-1} (see Fig. 3a), indicating that the repeating unit in Fig. 1c obtains 2.3 electrons and 1.15Zn^{2+} while discharging. Based on the XPS Cu 2*p* (Fig. 4c) and the capacity loss after a self-discharge test (inset of Fig. 6d), we assume that the additional capacity of 39 mAh g^{-1} comes from the redox region of Cu^{2+}/Cu^{+}. This capacity of 39 mA g^{-1} corresponds to accepting about 0.3 electrons, and thus the 0.3 Cu^{2+} in the repeating unit in its pristine state (left side, Fig. 1c) reduced at the discharged state (right side, Fig. 1c). Because $Cu_3(HHTP)_2$ consists of three repeating units of the structural formula in Fig. 1c, the overall reaction formula of Zn-$Cu_3(HHTP)_2$ battery is as follows:

$$Cu_3(HHTP)_2 + 3.45Zn^{2+} + 6.9e^{-} \rightarrow Zn_{3.45}[Cu_3(HHTP)_2]$$

Supplementary Note 3: Analysis of Charge Storage Mechanism

In order to distinguish the fraction of the current arising from cation intercalation and that from capacitive processes at specific potentials, responsive current (i) at a fixed potential (V) can be described as the combination of two separate mechanisms, namely capacitive effects ($k_1\nu$) and diffusion-controlled insertion ($k_2\nu^{1/2}$) according to:

$$\boldsymbol{i(\nu) = k_1\nu + k_2\nu^{1/2}} \qquad \text{(Supplementary Equation 2)}$$

where ν is the sweep rate. By separating the diffusion-controlled process where i varies as $\nu^{1/2}$, and the capacitive process where i varies as ν, the equation of Supplementary Equation 2 is rearranged to:

$$\boldsymbol{i(\nu)/\,\nu^{1/2} = k_1\nu^{1/2} + k_2} \qquad \text{(Supplementary Equation 3)}$$

Supplementary References

1. Zhang, L., Chen, L., Zhou, X. & Liu, Z. Towards high-voltage aqueous metal-ion batteries beyond 1.5 V: The zinc/zinc hexacyanoferrate system. *Adv. Energy Mater.* **5**, 1400930 (2015).

2. Zhang, L., Chen, L., Zhou, X. & Liu, Z. Morphology-dependent electrochemical performance of zinc hexacyanoferrate cathode for zinc-ion battery. *Sci. Rep.* **5**, 18263 (2015).

3. Trócoli, R. & La Mantia, F. An aqueous zinc-ion battery based on copper hexacyanoferrate. *ChemSusChem* **8**, 481−485 (2015).

4. Weppner, W. & Huggins, R. A. Determination of the kinetic parameters of mixed-conducting electrodes and application to the system Li_3Sb. *J. Electrochem. Soc.* **124**, 1569−1578 (1977).
